# Commercially available kelp and seaweed products – valuable iodine source or risk of excess intake?

**DOI:** 10.29219/fnr.v65.7584

**Published:** 2021-03-30

**Authors:** Inger Aakre, Dina Doblaug Solli, Maria Wik Markhus, Hanne K. Mæhre, Lisbeth Dahl, Sigrun Henjum, Jan Alexander, Patrick-Andre Korneliussen, Lise Madsen, Marian Kjellevold

**Affiliations:** 1 Department of Seafood and Nutrition, Institute of Marine Research, NO-5817 Bergen, Norway; 2 Department of Clinical Science, Faculty of Medicine, University of Bergen, Bergen, Norway; 3 Nofima, Norwegian Institute of Food, Fisheries and Aquaculture Research, Ås, Norway; 4 Department of Nursing and Health Promotion, Oslo Metropolitan University (OsloMet), Oslo, Norway; 5 Division of Infection Control, Environment and Health, Norwegian Institute of Public Health, Oslo, Norway; 6 Department of Biology, University of Copenhagen, Copenhagen Ø, Denmark

**Keywords:** iodine, recommended intake, tolerable upper intake level, seaweed, kelp, macroalgae, iodine excess, novel food

## Abstract

**Background:**

Seaweeds and kelps, also known as macroalgae, have long been common in the East-Asian diet. During recent years, macroalgae have entered the global food market, and a variety of macroalgae products are now available for consumers. Some macroalgae species are known to be particularly rich in iodine, but little data regarding the iodine content of macroalgae-containing foods exists.

**Objective:**

The aim of this research study was to analyse the iodine content in a large variety of commercially available macroalgae-containing foods and supplements and to evaluate whether such products are sources of adequate dietary iodine.

**Design:**

Ninety-six different products were collected after surveying the Norwegian market for commercially available macroalgae products, collected from three categories: 1) wholefood macroalgae products (*n* = 43), 2) macroalgae-containing foods (*n* = 39), and 3) dietary supplements containing macroalgae (*n* = 14). All products were analysed for iodine content by inductively coupled plasma-mass spectrometry (ICP-MS).

**Results:**

The iodine content in one portion of wholefood macroalgae products ranged from 128 to 62,400 μg. In macroalgae-containing foods, the iodine content ranged from 30 to 25,300 μg per portion, and in supplements it ranged from 5 to 5,600 μg per daily dose. The species with the highest analysed iodine content were oarweed, sugarkelp and kombu, with mean iodine levels of 7,800, 4,469 and 2,276 μg/g, respectively. For 54 products, the intake of one portion or dose would exceed the tolerable upper intake level (UL) for iodine.

**Discussion and conclusion:**

The iodine content in the included products was variable and for most products high, exceeding the tolerable upper intake level (UL) if consumed as a serving or portion size. The labelling of macroalgae species included, and declaration of iodine content, were inadequate or inaccurate for several products. As macroalgae-containing products are unreliable iodine sources, inclusion of such products in the diet may pose a risk of consuming excessive amounts of iodine.

## Popular scientific summary

- Concerns have been raised regarding the iodine content in edible macroalgae.
- We have collected commercially available macroalgae-foods, including whole food macroalgae, foods with macroalgae as an ingredient and supplements with macroalgae and analysed the iodine content.
- Highly variable levels of iodine were found between different macroalgae species and food products.
- Macroalgae-containing foods are an unreliable source of iodine as inclusion of such products in the diet may pose a risk of consuming excessive amounts of iodine.

Edible seaweeds, or macroalgae, are a part of the habitual diet in many East-Asian cultures (1–3). In Europe, macroalgae have traditionally been used by subpopulations in Iceland, the United Kingdom, and France (4, 5). However, during the recent decade, a variety of wholefood macroalgae and macroalgae-containing products have entered the global food market and are now available for European consumers (6). These products have gained increasing popularity in the western part of the world (5–7), and an important reason for the growing interest in dietary macroalgae is the expanding consumer view of macroalgae as healthy food or even as a ‘super-food’ (8–11).

Macroalgae are divided into three main groups: brown, red and green algae. Brown macroalgae (*Phaeophyceae*), such as kelps and perennials, are usually large in size, whereas the red (*Rhodophyceae*) and green (*Chlorophyceae*) macroalgae species are smaller (12). Macroalgae efficiently absorb inorganic compounds, such as minerals and other trace elements, from the seawater and utilise them for their metabolism. However, the internal structure and metabolism differ between the macroalgae groups, resulting in different absorption and accumulation rates. In addition, the absorption of minerals depends on a range of physical factors, such as availability of nutrients in the sea, oceanic currents, pH, salinity, temperature and solar irradiance. These factors vary with geographical location and conditions throughout the year, leading to geographical and seasonal variations in mineral content in the algae (13). Macroalgae are known to be particularly rich in iodine (14, 15), and brown algae, especially kelps, contain the highest amounts (15–17). In *Fucales* and *Laminariales*, the high level of iodine is due to the presence of haloperoxidases in the cell wall, facilitating uptake, conversion, and storage of iodine (18). These algae may thus contain amounts that are several orders of magnitude higher than in the surrounding water (18).

Iodine is an essential micronutrient required for the synthesis of thyroid hormones, thyroxine (T4) and triiodothyronine (T3). The thyroid hormones regulate a wide range of cellular and physiological functions, such as normal growth and development, neural differentiation, and metabolic regulation (19). Both iodine deficiency and iodine excess may lead to thyroid dysfunction, which may manifest itself as hypo- or hyperthyroidism (20–22). The consequences of inadequate iodine intake are well documented, and may lead to a wide spectrum of disorders, collectively known as iodine deficiency disorders (IDDs) (23). Documentation of health consequences resulting from excessive intake of iodine, however, is limited. The underlying mechanisms of the IDDs are related to inadequate thyroid hormone production and to disturbed action in the target tissues (24), and as a very high intake of iodine may result in thyroid dysfunction, the adverse health consequences of iodine deficiency may apply for iodine excess as well (25). IDDs may occur at all life stages and may cause several adverse health consequences, including goitre, increased infant mortality, impaired mental development, delayed physical development, increased prevalence of abortion and stillbirth, delayed puberty, and increased infertility (23, 26). In 2002, the Scientific Committee on Food established a tolerable upper intake level (UL) of 600 μg/day for adults and of 200 μg/day for children (27).

Iodine deficiency has been re-emerging in Europe (28), and mild-to-moderate iodine deficiency is reported in several population groups in Norway (29, 30). Iodine is present in only very few foods (31), and macroalgae may represent a new dietary iodine source. However, iodine levels over 8,000 μg/g are reported in some macroalgae species, and the intake of macroalgae-containing products may result in excessive iodine levels (14). Nevertheless, a maximum level of iodine has not been establised in such products (32).

The aim of this research study was to determine the iodine levels of macroalgae-containing products available in Norway and to evaluate whether these products are an adequate dietary iodine source. For the products with cooking instructions, the iodine content was analysed, and described before and after preparation.

## Methods

### Identification of products

**Fig. 1:**
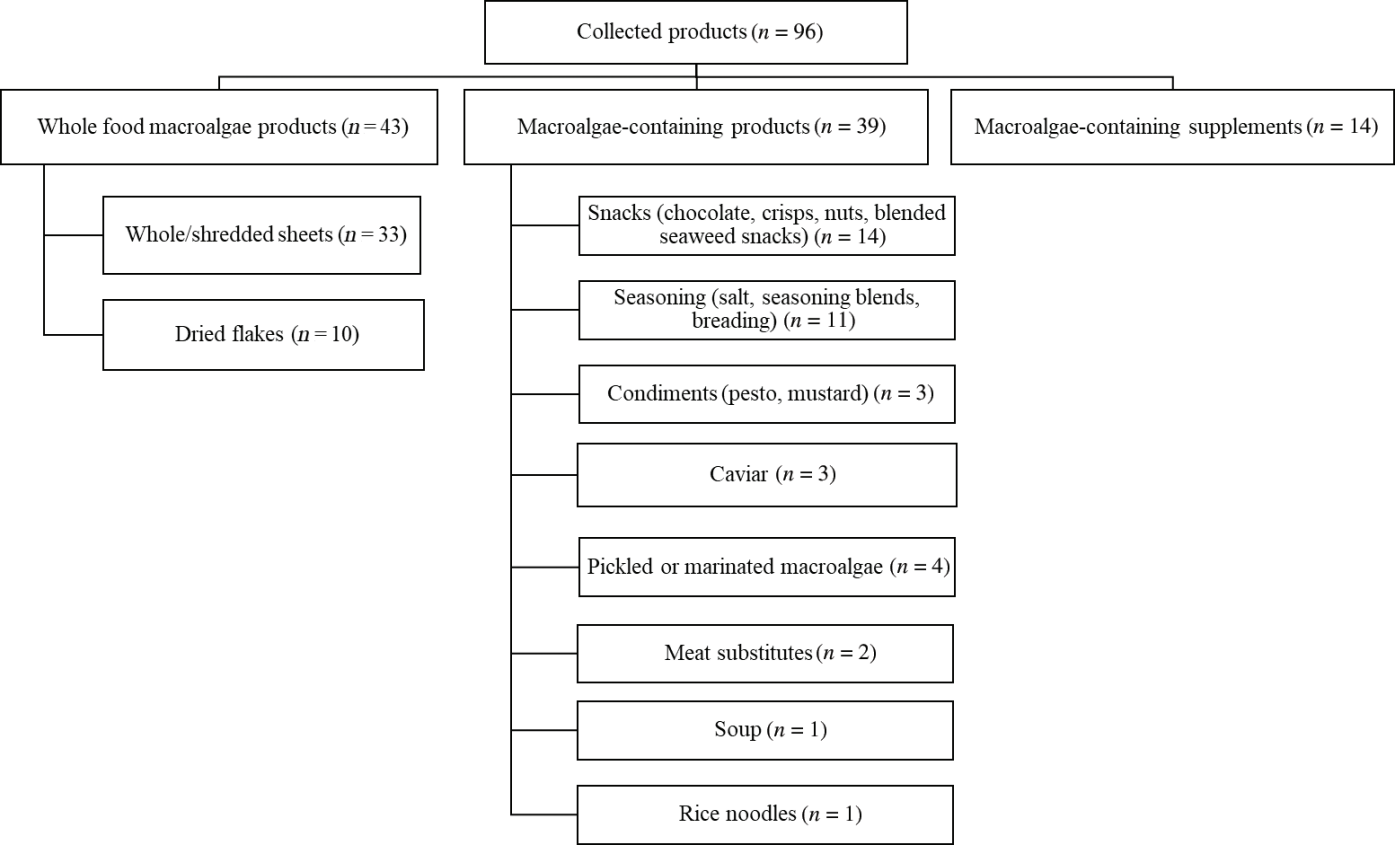
Overview of the collected macroalgae products in the different product categories.

The Norwegian market was surveyed to identify commercially available products containing macroalgae of marine origin from August to December 2019. Macroalgae products were selected from an in-store survey and an internet survey from three pre-selected main categories: [1] products with macroalgae as the only ingredient (wholefood macroalgae), [2] products with macroalgae as an ingredient (macroalgae-containing foods), and [3] macroalgae-containing dietary supplements. Sushi was excluded from the market survey, as sushi and nori, which are the sheets that are typically used to wrap sushi rolls, had already been collected and analysed by the Norwegian Food Safety Authority (NFSA) (33). In total, 96 products were collected, and an overview of the collected products is shown in different product categories Fig. 1.

The in-store survey was limited to the two largest cities in Norway, Oslo and Bergen. We aimed at selecting products that were available at a large scale if possible. Norwegian online stores were found by searching with the following keywords: ‘seaweed’, ‘kelp’ and ‘algae’, as well as the corresponding words in Norwegian. Three separate items, preferably with three separate batch numbers, of each identified product were purchased. If different batch numbers were not possible to obtain, we selected products with different best before dates (BBDs). If neither different batch numbers nor BBDs were available, three items were conveniently selected.

The following information labelled on the product packaging was recorded: type of macroalgae (common name), macroalgae species (Latin name), compliance with language requirements for labelling of products, declared iodine content and the recommended dose for supplements.

### Preparation procedure of products

For each product, equal quantities were drawn from the three collected items of the product to form a homogenised composite sample. A subsample of 20–50 g was taken from the composite sample, freeze-dried and re-homogenised before being portioned in appropriate amounts for analysis. Supplements (*n* = 14) and products rich in fat (*n* = 6), including caviar (*n* = 3), pesto (*n* = 2) and mustard (*n* = 1), were not freeze-dried.

### Preparation procedure of products with cooking instructions

Thirty products displayed specific cooking instructions on the product packaging. These products were prepared accordingly with the purpose of comparing the iodine content before and after cooking. The cooking processes were not carried out in the sense of accounting for the loss of iodine through different stages of treatment, but from a consumer perspective where the goal was to examine the products’ iodine content after preparation according to the recommended processing method. Of the 30 processed samples, 21 consisted of processed macroalgae, 6 consisted of processed macroalgae and broth, and three samples consisted of only broth. Water samples were extracted from the boiling pot using a pipette after hydrothermal preparation of the macroalgae. When multiple preparation suggestions were provided, rehydration was chosen. Where different heat treatments were suggested, boiling was preferred over baking in the oven. A detailed description of the different cooking methods is provided in Appendix 1. Twenty-seven of the processed samples consisted of a composite sample of three items of the products, the remaining consisted of two items (*n* = 2) or one item (*n* = 1) of the products.

### Iodine determination by inductively coupled plasma mass-spectrometry

Iodine was determined by inductively coupled plasma-mass spectrometry (ICP-MS), at the Institute of Marine Research, an accredited laboratory according to NS-EN ISO 17025. Samples were prepared by adding tetramethylammonium hydroxide, #x03B1;-amylase and water to the freeze-dried sample. #x03B1;-Amylase was added to food products with macroalgae as an ingredient (macroalgae-containing products) in order to hydrolyse the starch. Extraction at 90°C for 3 h followed. Composite samples were analysed with one analytical replicate per sample. Individual samples of water were analysed. For the recollected products, individual samples of three replicates were studied. This was carried out by trained laboratory technicians.

The limit of quantification (LOQ) for the method was calculated as 10 times the standard deviation (SD) from 20 blind samples analysed on the same day, and was estimated to be 0.04 mg/kg dry weight for dry samples and 0.32 μg/L in aqueous solutions. Measurement uncertainty was 20% for concentrations >10 LOQ. The analysed values of standard reference materials are shown in Table 1. All iodine values were calculated from freeze-dried samples back to the original sample and are, therefore, considered as wet weight (w.w.) unless otherwise stated.

**Table 1:** Iodine content (mg/kg) in certified reference material in comparison with the analysed and measured values

Reference material	Certified value	Analysed	Control chart	RSD (%)
Fish muscle (ERM-BB 422)	1.4 ± 0.4	1.29 ± 0.03 (*n* = 6)	1.26 ± 0.06 (*n* = 56)	2.52^a^ 4.33^b^
Kelp powder (3232 NIST)	944 ± 88	*	798 ± 73.10 (*n* = 27)	9.16^b^

RSD, relative standard deviation. Values are presented as means ± standard deviation, expressed as milligrams per kilogram

* Currently not used as a control material due to stability issues.

a Analysed.

b Measured.

### Data management

We assessed the contribution of different products to the daily recommended intake (RI) for adults of 150 μg/day and the risk for exceeding the UL of 600 μg/day for adults using the Nordic Nutrition Recommendations (NNRs) (34). For wholefood macroalgae products, a portion size of 8 g was applied as suggested by others (7). For foods containing macroalgae, we used standard portion sizes from the Norwegian report, ‘Weights, measures and portion sizes for foods’ (35). If standard portion sizes were not found, we used a unit or an otherwise suited measure. All portion sizes used are described in Appendix 2. For supplements, the manufacturers’ recommended daily dose (i.e. grams, milligrams, capsule, tablet, teaspoon and knife blade tip) was used to calculate the relative contribution to RI and whether the recommended dose exceeded the UL. For one powdered supplement, the quantity was provided in teaspoons, and as there is no standard weight for different types of macroalgae powder, the dose recommended for a powder product by a different manufacturer was used. The recommended dosage and the calculations are described in Appendix 3.

Descriptive statistics were performed in the statistical software IBM Statistical Package for the Social Sciences (SPSS), version 25. For macroalgae wholefood products and macroalgae-containing foods, data are presented as means ± SD per gram of composite samples.

## Results

The type of store where the products were purchased and their country of origin is tabulated in Table 2. Sixty-nine per cent of the products were obtained from stores located in Bergen or Oslo, and 31% of the products were bought from online shops. Thirty-nine per cent of the products originated from Norway, whereas 19, 10, 10 and 20% of the products originated from Denmark, United Kingdom, South Korea or other countries, respectively.

**Table 2:** Procurement information and country of origin of the sampled commercially available macroalgae products (*n* = 96)

Procurement information	*n*	%^b^
Type of store^a^	66	69
Health food stores	39	41
Global food stores	14	15
Fish markets	8	8
Grocery chain stores	3	3
Other stores	2	2
Internet	30	31
**Country of origin**		
Norway	37	39
Denmark	18	19
United Kingdom	10	10
South Korea or Korea	10	10
Sweden	6	6
China	4	4
France	2	2
Thailand	2	2
Germany	2	2
Italy	1	1
Switzerland	1	1
United States	1	1
Taiwan	1	1

a Fifty-one products were purchased from stores located in Bergen, 14 in Oslo and 1 from stores in both Oslo and Bergen.

b All percentages are calculated from the total number (96).

Table 3 shows the iodine content (μg/g) in the three main product categories (wholefood macroalgae, foods containing macroalgae and supplements containing macroalgae) and their subcategories. The wholefood products had the highest iodine content, with a mean iodine content of 1,319 μg/g, and the dried macroalgae flakes come under the subcategory with the highest iodine content with a mean value of 2,528 μg/g. The most iodine-rich product with a maximum value of 12,000 μg/g belonged to this category. The mean iodine content in foods containing macroalgae was 184 μg/g, and the iodine content in the different subcategories were highly variable, with mean values ranging from 1 to 611 μg/g. The subcategory with the highest iodine content within the category of foods containing macroalgae was seasoning blends, where the maximum iodine content was 2,500 μg/g. The mean iodine content in the 14 supplements was 244 μg/g.

**Table 3:** Iodine content (μg/g) in the different categories of commercially available macroalgae containing products (*n* = 96)

Products	Iodine content in μg/g		
	Mean ± SD	Median (p25–p75)	Min–Max
Wholefood macroalgae (*n* = 43)	1,319 ± 2,483	200 (92–1,300)	5–12,000

Whole/ shredded sheets (*n* = 33)	952 ± 1,978	190 (50–410)	5–8,100
Dried flakes (*n* = 10)	2,528 ± 3,571	1,300 (773–2,900)	140–12,000

Foods containing macroalgae (*n* = 39)	184 ± 505	20 (2–50)	0.3–2,500

Snacks (chocolate, crisps, nuts and blended seaweed snacks) (*n* = 14)	23 ± 22	20 (5–28)	2–86
Salt or seasoning blends, breading (*n* = 11)	611 ± 829	130 (29–1,400)	2–2,500
Condiments (mustard, pesto) (*n* = 3)	33 ± 24	44 (NA)	6–50
Pickled or marinated macroalgae (*n* = 4)	12 ± 20	2 (1–32)	0.3–42
Meat substitutes, rice noodles, soup (*n* = 4)	2	1 (1–4)	1–5
Caviar (*n* = 3)	1	1 (NA)	0.6–1

Supplements containing macroalgae (*n* = 14)	244 ± 179	260 (48–383)	8–560

All iodine values are presented in μg/g of original sample (wet weight).

Table 4 shows how one portion of the different wholefood products according to species and one serving size of the different food categories contribute to the RI (150 μg/day) for iodine and whether the UL (600 μg/day) was exceeded. All wholefood products had a substantial contribution to the RI, where a portion of the least iodine-rich product, consisting of truffle seaweed, contributed to 85% of the RI. A large share of the wholefood products (31 of 40) exceeded the UL for iodine, and some of the products by manyfold. Sugar kelp and oarweed were the two species with the highest iodine content and the UL would be exceeded 59 and 104 times, respectively, by intake of one portion. Three products in the category of ‘flakes’ were sold in spice grinders and excluded from the portion estimates for the wholefood products. These included the species dulse, sugar kelp and mixed species, which contained 180, 4,700 and 2,300 μg iodine per gram, respectively. However, consuming only one gram of the sugar kelp and mixed species flakes from the spice grinders would exceed the UL by 7.8 and 3.8 times, respectively. The macroalgae-containing foods also had a substantial contribution to the RI, where one portion of the least iodine-rich products, which were caviar and rice noodles, contributed with 20 and 57%, of the RI, respectively. Twenty-one out of the 39 macroalgae-containing foods would exceed the UL if consuming one portion size. For salt and seasoning blends, a daily intake of 6 g was used in the calculations. The two food products with the highest iodine contribution from one portion included chocolate and breading.

**Table 4:** One portion of the different macroalgae products’ contribution to the RI and UL of iodine

Wholefood macroalgae	Iodine content						
Species	*n*^e^	Mean^a^ (μg/g)	SD (μg/g)	Min-max (μg/g)	Iodine per portion^b^ (μg)	% of RI^c^	Times UL^d^
Irish moss or carrageen moss	1	16	NA	NA	128	85	0.2
Nori	2	18	4	15–21	144	96	0.2
Sea spaghetti^f^	6	42	23	5–72	336	224	0.6
Dulse	3	96	47	47–140	768	512	1.2
Bladder wrack	1	120	NA	NA	960	640	1.6
Wakame	5	172	63	92–260	1,376	917	2.3
Toothed wrack	2	220	42	190–250	1,760	1,173	2.9
Arame or sea oak	1	400	NA	NA	3,200	2,133	5.3
Winged kelp	5	552	402	190–990	4,416	2,944	7.4
Mix	4	740	648	140–1,300	5,920	3,947	9.9
Truffle seaweed	1	1,400	NA	NA	11,200	7,467	19
Kombu	3	2,267	945	1,200–3,000	18,136	12,091	30
Sugar kelp^f^	4	4,400	5,222	300–12,000	35,200	23,467	59
Oarweed	2	7,800	424	7,500–8,100	62,400	41,600	104

Foods containing macroalgae	Iodine content						
Product categories	*n*	Mean μg/g	SD	Min-max	Iodine per portion (μg)	% of RI	Times UL^d^

Caviar	3	1	NA	NA	30	20	0.1
Rice noodles with seaweed	1	1	NA	NA	85	57	0.1
Blended snack	3	17	7	9–23	136	91	0.2
Mustard	1	6	NA	NA	96	64	0.2
Meat substitutes	2	1.2	1	0.7–1.7	180	120	0.3
Nori-snack	4	25	7	16–32	200	133	0.3
Crisps	2	11	13	2–20	264	176	0.4
Nuts	2	25	27	6–44	500	333	0.8
Pesto	2	47	4	44–50	846	564	1.4
Marinated or pickled seaweed	4	11	20	0.3–42	1,100	733	1.8
Soup	1	5	NA	NA	1,750	1,167	2.9
Seasoning blends	5	368	634	25–1,500	1,840	1,227	3.1
Salt	4	588	594	2–1,400	2,940	1,960	4.9
Chocolate	3	31	48	2–86	3,100	2,067	5.2
Breading	2	1,265	1,747	29–2,500	25,300	16,867	42

a The mean value was calculated for compiled products (two or more). All single values are obtained from measurements in pooled samples comprising three subsamples for each product.

b A portion size of 8 g was used for all dried wholefood macroalgae products (7). For foods containing macroalgae, standard portion sizes have been used.

c RI: Recommended Intake for adults from the Nordic Nutrition Recommendations (NNR) of 150 μg/ day (34).

d UL: Tolerable upper intake level for adults from NNR of 600 μg/day (34).

e Three products were excluded from the portion estimations from the wholefood category, one product with dulse, one product with sugar kelp and one product with mixed species, as these were sold in a spice grinder.

f One product includes fresh algae.

The contribution of the recommended daily dose of each macroalgae-containing supplement to the RI and UL is tabulated in Table 5. Two for the supplements had a very low iodine content, and the recommended doses contributed to only 3 and 8% of the RI. At the same time, the recommended dose for four products exceeded the RI, and two products would lead to an iodine intake exceeding the UL for iodine. Most of the supplements had a substantially lower iodine content than the content declared on the product packaging. For the supplements with an iodine declaration, all analysed iodine values were lower than the declared content, except for one product.

**Table 5:** The recommended dose of each macroalgae-containing supplements’ contribution to the RI and UL of iodine

Supplement type	Species *n* supplement	Declared content per proposed dose (μg)	Analysed iodine content per recommended dose (μg)	RI (%)^a^	Times UL^b^	Difference^c^ Direction (%)
Capsules	Oarweed	200	5	3	0.0	↓ (97.5)
Tablets	Rockweed, Wakame	None	12	8	0.0	NA
Capsules	Bladderwrack	194	78	52	0.1	↓ (60)
Tablets	Giant Kelp	200	40	27	0.1	↓ (80)
Tablets	Rockweed, Bladderwrack	225	90	60	0.1	↓ (60)
Tablets	Rockweed, Bladderwrack	150	32	21	0.1	↓ (79)
Powder	Rockweed, Bladderwrack, Toothed Wrack	None	80	53	0.1	NA
Tablets	Not identified	200	64	43	0.1	↓ (68)
Capsules	Rockweed	150	116	77	0.2	↓ (23)
Capsules	Bladderwrack	150	144	96	0.2	↓ (4)
Powder	Sea Lettuce	None	310	207	0.5	NA
Powder	Sea Lettuce	None	540	360	0.9	NA
Capsules	Rockweed	150	810	540	1.4	↑ (440)
Powder	Rockweed	None	5,600	3,733	9.3	NA

a RI: Recommended Intake for adults from the Nordic Nutrition Recommendations (NNR) of 150 μg/day (34).

b UL: Tolerable upper intake level for adults from NNR of 600 μg/day (34).

c Direction of the difference between the declared iodine and analysed content indicated by an ↑↓ arrow using declared content as a reference. All single values consist of pooled samples of three subsamples for each product.

**Fig. 2:**
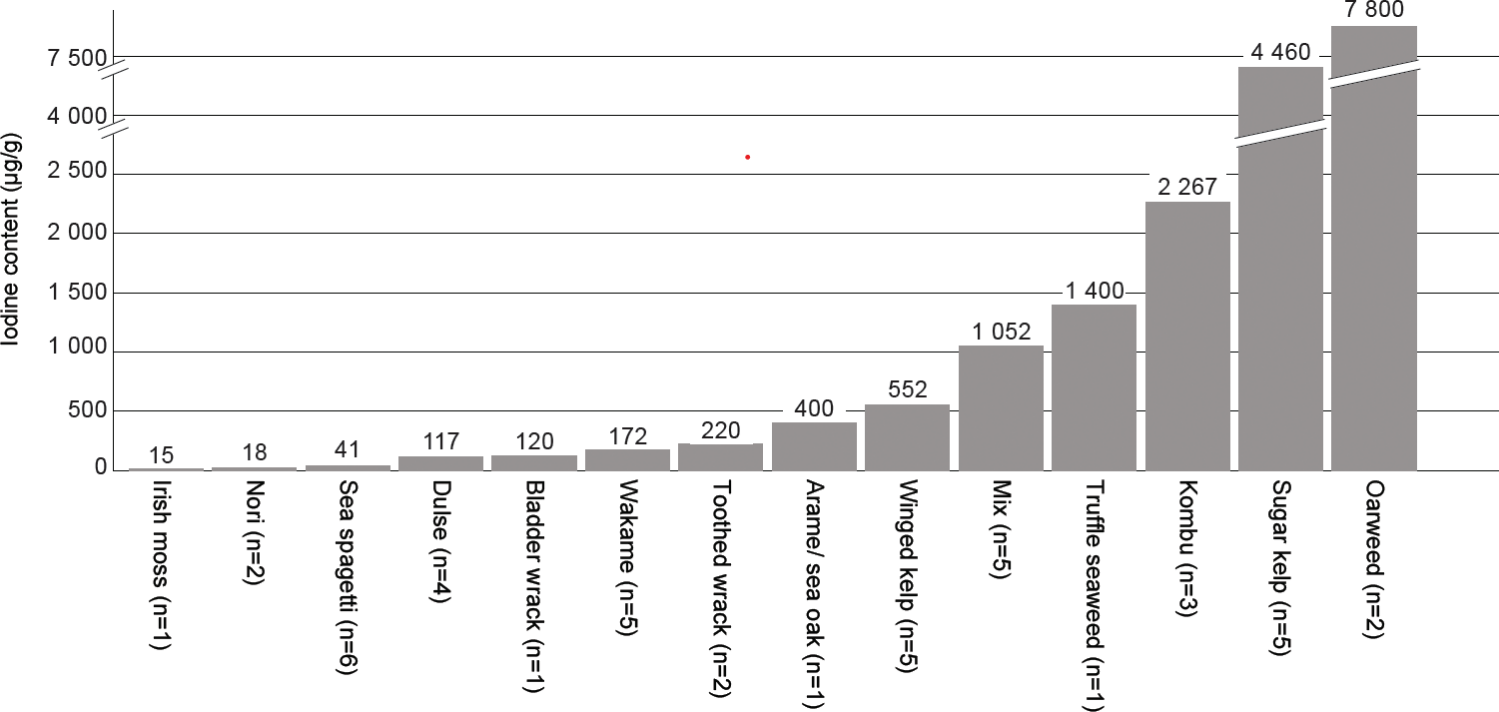
Mean iodine content (μg/g) in commercially available wholefood macroalgae products according to species (*n* = 43).

In the collected products, 17 different species of macroalgae were identified. The most commonly found species were sugar kelp *(Saccarina latissima)*, winged kelp *(Alaria esculenta)* and wakame *(Undaria pinnatifida)*, all from the group of brown algae. In total, species from the group of brown algae *(Phaeophyceae)* were present in 62% (*n* = 59) of the collected products, while red algae *(Rhodophyta)* were present in 18% (*n* = 17). Thirteen (14%) products contained a mixture of different brown algae species or different groups (e.g. brown and red, green and red). Green algae were infrequently found in 2% (*n* = 2) of the products. Table 6 shows the iodine content within the different product categories according to the groups: brown, red, green and mixed algae. Within all the three product categories the products containing brown macroalgae reported the highest iodine content. Within the product category of wholefood products, oarweed *(Laminaria digitata)*, sugar kelp and kombu *(Laminaria japonica)* had the highest iodine content, with mean values of 7,800, 4,460 and 2,267 μg/g, respectively (Fig. 2).

**Table 6:** Iodine content (μg/g) in the different groups (brown, red and green) of macroalgae

Different groups of microalgae	Iodine content (μg/g)		
Wholefood macroalgae (*n* = 43)	Mean ± SD	Median (p25–p75)	Min-Max
Brown algae^a^ (*n* = 30)	1,651 ± 2,884	255 (113–2,075)	5–12,000
Red algae^b^ (*n* = 8)	240 ± 473	73 (17–170)	15–1,400
Mixed algae^c^ (*n* = 5)	1,052 ± 895	1,300 (180–1,800)	140–2,300

Foods containing macroalgae (*n* = 39)			
Brown algae^d^ (*n* = 24)	276 ± 627	25 (2–94)	0.7–2,500
Red algae^e^ (*n* = 9)	57 ± 110	25 (13–29)	5–350
Mixed algae^f^ (*n* = 2)	21 ± NA	21 (NA)	0.3–42
Unidentified (*n* = 4)	6 ± 10	1 (0.7–15)	0.6–20

Supplements containing macroalgae (*n* = 14)			
Brown algae^g^ (*n* = 7)	294 ± 184	290 (160–450)	8–560
Green algae^h^ (*n* = 2)	43 ± NA	43 (NA)	31–54
Mixed algae^I^ (*n* = 4)	204 ± 143	235 (58–320)	27–320
Unidentified (*n* = 1)	NA	NA	460

All iodine values are presented in μg/g of the original sample (wet weight).

a Oarweed, kombu, sugar kelp, arame, bladderwrack, toothed wrack, wakame, winged kelp and sea spaghetti.

b Dulse, nori.

c Oarweed, sugar kelp, winged kelp and dulse (*n* = 1); dulse, sea lettuce and nori (*n* = 1); sugar kelp, winged kelp, dulse and nori (*n* = 1); sugar kelp, winged kelp and dulse (*n* = 1); and wakame, kombu and nori (*n* = 1).

d Oarweed, sugar kelp, winged kelp, hijiki, wakame, sea spaghetti, bladderwrack, toothed wrack and rockweed.

e Dulse, nori and truffle seaweed.

f Oarweed, sugar kelp, dulse and winged kelp (*n* = 1); sea spaghetti and dulse (*n* = 1).

g Rockweed, bladderwrack, giant kelp and oarweed.

h Sea lettuce.

i Rockweed and bladderwrack (*n* = 2), rockweed, bladderwrack and toothed wrack (*n* = 1); rockweed and wakame (*n* = 1).

Different products were labelled according to the common name and species at a varying accuracy. For 12 products, neither the common name nor the Latin name of the species was included on the package insert. For seven products, the species were identified through oral communication with the manufacturers, and for the remaining five products, the species included were unidentified. The packaging was labelled with both the common and Latin name for 54% (*n* = 52) of the products. Moreover, 28% (*n* = 27) of the products provided only the common name, whereas 5% (*n* = 5) of them stated only the Latin name. For three products, the common and Latin name did not match.

Of the wholefood products, 21 declared the iodine content, whereas 22 did not. For the foods containing macroalgae, only three of 39 products declared the iodine content. For these, the analysed values were lower than the declared (Table 7). Foods containing macroalgae (*n* = 3) reported the mean declared iodine content to be 101 μg/g, while the analysed values were 76 μg/g. For the wholefood macroalgae products (*n* = 21), the declared content of iodine was significantly lower than the analysed values, with mean iodine levels of 553 and 1,713 μg/g for declared and analysed values, respectively. For the majority of the macroalgae products, the analysed values were markedly different from the declared values. For 14 of the products, the analysed iodine content was lower than the declared content, while for 10 products, the analysed iodine content was higher than the declared content.

**Table 7:** Analysed and declared iodine content of commercially available macroalgae products (*n* = 24)

	Declared iodine content (μg/g)^a^	Analysed iodine content (μg/g)^a^	Difference^b^ direction (%)	*P*^c^
**Wholefood macroalgae**				
Arame or sea oak	978	400	↓ (59)	

Kombu	4	1,200	↑ (29,900)	

Oarweed	29	8,100	↑ (27,831)	
	3,360	7,500	↑ (123)	

Sea spaghetti	4	72	↑ (1,700)	
	3,360	50	↓ (99)	
	111	44	↓ (60)	
	2	28	↑ (1,300)	

Sugar kelp	64	12,000	↑ (18,650)	
	50	3,400	↑ (6,700)	

Toothed wrack	456	250	↓ (45)	

Wakame	17	140	↑ (724)	

Winged kelp	1,300	990	↓ (24)	
	1,300	970	↓ (25)	
	459	190	↓ (59)	
	2	190	↑ (9,400)	

Total brown macroalgae (*n* = 16)	719 ± 1,128	2,220 ± 3,672	↑ (209)	<0.001

Dulse	140	16	↓ (89)	
	100	4	↓ (96)	
	47	46	↓ (2)	

Nori	15	4	↓ (73)	

Total red macroalgae (*n* = 4)	75 ± 56	18 ±20	↓ (76)	NA

Mixed species	52	140	↑ (169)	

Total all species (*n* = 21)	553 ± 1,023	1,713 ± 3,313	↑ (210)	<0.001

**Foods containing macroalgae**				
Breading	111	96	↓ (14)	
	95	87	↓ (8)	

Snacks	96	44	↓ (54)	

Total foods with macroalgae (*n* = 3)	101 ± 9	76 ± 28	↓ (25)	

a All values are presented as the analysed or declared value for the specific product in μg/g. All single values are obtained from measurements in pooled samples comprising three sub-samples for each product. For compiled data, values are expressed as mean ± SD in μg/g.

b Direction of the difference between the declared iodine and analysed content is indicated by an ↑↓ arrow using the declared content as a reference.

c Tested by one-sample Kolmogorov–Smirnov test.

The values of iodine content in unprocessed samples and in samples prepared according to the cooking instructions from the manufacturer are presented in Table 8. For the samples where consumption of the broth was recommended, the iodine content in water was analysed as well. For most products, the iodine content was reduced on a dry weight basis after cooking. The iodine concentration in water was varying, ranging from 28 and up to as much as 290,000 μg/L.

**Table 8:** Iodine content in commercially available macroalgae products pre- and post-preparation^a^ (*n* = 30)

Common name	Type of product	Iodine content pre-preparation (μg/g)^b^	Iodine content d.w. pre-preparation (μg/g)^c^	Iodine content post preparation (μg/g)^d^	Iodine content d.w. post preparation (μg/g)^e^	Iodine concentration post-preparation in water (μg/L)^f^
Sea spaghetti	Whole dried macroalgae^i^	28	31	6	49	
	Whole dried macroalgae^i^	50	53	15	44	
	Whole dried macroalgae^i^	72	77	2	28	
	Whole dried macroalgae^i^	50	56	4	32	
	Whole fresh macroalgae^i^	5	12	7	52	

Sugar kelp	Whole dried macroalgae	1,900	2,120	59	474	34,000
	Whole fresh macroalgae^i^	300	836	160	1,406	

Dulse	Whole dried macroalgae	140	152	23	136	
Oarweed	Whole dried macroalgae	8,100	8,697	160	1,444	290,000
	Whole dried macroalgae	7,500	8,545	140	901	

Winged kelp	Whole dried macroalgae	190	216	4	54	
	Whole dried macroalgae	190	215	10	159	

Arame or sea oak	Whole dried macroalgae	400	434	33	213	

Bladder wrack	Whole dried macroalgae	120	137	4	23	

Toothed wrack	Whole dried macroalgae	250	271	50	182	
	Whole dried macroalgae	190	221	11	77	

Irish moss	Whole dried macroalgae	-^g^	-^g^	16	139	

Wakame	Whole dried macroalgae^i^	140	152	3	70	1,100
	Whole dried macroalgae^i^	260	294	17	186	
	Whole dried macroalgae^i^	200	216	19	244	2,100
	Noodles	0,9	1	0,2	1	
	Whole dried macroalgae^i^	170	183	11	217	1,200
	Whole dried macroalgae^i^	92	98	9	120	
	Soup	5	5	-^h^	-^h^	28

Kombu	Whole dried macroalgae	2,600	2,704	-^h^	-^h^	120,000
	Whole dried macroalgae	3,000	3,257	88	813	130
	Whole dried macroalgae	1,200	1,273	-^h^	-^h^	

Nori	Whole dried macroalgae	15	16	2	24	64
	Whole dried macroalgae	21	24	2	29	

Mix	Whole dried macroalgae^i^	220	245	19	197	

d.w. = dry weight. All single values are obtained from measurements in pooled samples comprising three sub-samples for each product.

a Detailed description of the processing methods and dry weight numbers are provided in Appendix 1.

b Iodine content pre-preparation in μg/g wet weight.

c Iodine content pre-preparation in μg/g dry weight (freeze dried sample).

d Iodine content in μg/g wet weight after preparing the products according to the packaging insert.

e Iodine content in μg/g dry weight (freeze dried sample) after preparing the products according to the packaging insert.

f Iodine concentration in water in μg/L for the products where the cooking instructions recommended using the boiling water as broth.

g Pre-analysis was not conducted due to homogenisation issues.

h Only the broth was analysed.

i Sold as shredded.

## Discussion

This research study presents analysed values of iodine content in a wide range of commercialised macroalgae food products and supplements. We found a significant variation in the iodine content between the different products of macroalgae. The iodine content in one portion of wholefood macroalgae ranged from 128 to 62,400 μg, the iodine content per portion in macroalgae-containing foods ranged from 30 to 25,300 μg and in supplements the iodine content per daily dose ranged from 5 to 5,600 μg. Several products had inadequate labelling of species included and iodine content. The species with the highest analysed iodine content were oarweed, sugar kelp and kombu, with mean iodine values of 7,800, 4,469 and 2,276 μg/g, respectively.

The wholefood macroalgae products reported the highest iodine content, with a mean iodine level of 1,319 μg/g, compared with that of 184 μg/g in foods with macroalgae. Consumption of one portion (8 g) (7) of the different wholefood macroalgae products would provide a higher intake of iodine than UL for 31 of 40 products. The species included in the products which were not exceeding the UL were Irish moss (*Chondrus crispus*), truffle seaweed, nori (*Porphyra spp*.) and sea spaghetti (*Himanthalia elongate*). For the remaining species, as well as the mixed-species products, the UL would be exceeded by at least 1.6 times, and as many as 30, 59 and 104 times by kombu, sugar kelp and oarweed, respectively, by intake of one portion. Macroalgae-containing foods with moderate-to-low iodine levels may be used as an alternative iodine source, for example, by those who exclude or limit lean fish or dairy from the diet, and one portion of a macroalgae-containing food could contribute substantially to the RI for iodine. However, for most of the macroalgae-containing foods included in this study, one portion would exceed the RI and for several foods also the UL. For many of the wholefood products and the foods containing macroalgae, one portion would also result in an iodine intake exceeding the lowest observed adverse effect level (LOAEL) of 1,800 μg/day (27). Thus, frequent consumers of these products may be at risk of adverse health consequences caused by excessive iodine intakes. This is in accordance with another study, where the estimated iodine content of commercial macroalgae products is estimated to be 6,000 μg/g, and the estimated iodine content per serving was exceeding 10,000 μg for several products (6). Excessive iodine status was observed in a study of Norwegian macroalgae consumers, where the median UIC was 1,200 μg/L, and the estimated iodine intake from macroalgae was 2,200 μg per day (36). A long-term excessive iodine intake has been associated with hypothyroidism, goitre, autoimmune thyroid disease and iodine-induced hyperthyroidism in epidemiological studies (37, 38). An excessive iodine intake has also been associated with adverse effects on foetal growth (39). Thyroid disorders caused by an excessive iodine intake from dietary macroalgae have been described in different population groups, such as neonates (40), breastfed infants (41), school aged children (42), and adults (43, 44). Still, the mechanisms of excessive iodine intake and its consequences on health remain an area of limited research, and further studies are warranted.

We found that kelps of the brown algae group represent the most concentrated sources of iodine, which has been confirmed by others (15, 16); however, large variations were observed both within and between species in the wholefood macroalgae products. A US study from 2004 analysed a large amount of commercially available seaweeds, including 12 different species (14). They found a highly variable iodine content, ranging from 16 μg/g in nori to over 8,000 μg/g in kelp. Some of the wholefood products with the largest intra-species variation of iodine in this study included were winged kelp (190–990 μg/g), kombu (1,200–3,000 μg/g) and sugar kelp (300–12,000 μg/g). A high intra-species variation in sugar kelp has also been reported by others, and in a study from Norway, the iodine content varied from 1,556 to 7,208 μg/g dry weight (17). Possible reasons for high intra-species variability may be due to different life stages of the thallus or different parts of the thallus included in the different analysed samples (17). However, as these samples were store-bought and ready to consume, post-harvest storage conditions may have affected the iodine content, as iodine may be reduced with time when stored in open containers, particularly under humid conditions (14). Large variability was also seen for the iodine content in and between the different food categories, where, for instance, iodine content in nuts varied from 6 to 44 μg/g and in breading from 29 to 2,500 μg/g. The large variability in iodine between and within product categories in this study is likely attributed to different quantities of macroalgae included in the products and/or different species included. However, the quantity of macroalgae was rarely listed in the products’ ingredient list, and therefore, it is impossible to conclude whether the quantity or the species led to these large variations. Variations in iodine content are also found in conventional foods, such as lean fish, processed fish (i.e. fish burgers) and dairy products (45, 46); however, this was to a smaller degree compared with the macroalgae products in this study.

For the 14 analysed supplements, the iodine content varied from 5 to 5,600 μg per dose. Similarly, variations in the iodine content of UK-retailed macroalgae-containing supplements ranged from 210 to 3,840 μg per recommended dose (47). The declared iodine content per recommended dose deviated with more than the measurement uncertainty for most supplements, indicating that the declared values are not reliable. Based on the highly variable iodine content in macroalgae-containing supplements, such supplements may pose a health risk. Others have advised against the use of macroalgae-containing dietary supplements, and a review of iodine supplement use in pregnancy recommended that women who are pregnant, lactating or planning to conceive should refrain from using such supplements (48). Furthermore, the American Thyroid Association advise against consuming an iodine or kelp supplement containing >500 μg iodine daily for all individuals (49).

The labelling of the macroalgae products included in this study was variable, and for 12 products, the product packaging did not provide any information regarding the type of macroalgae. Similarly, a study from United Kingdom, which included 226 different macroalgae products, found that 16% did not include labelling of any sort (6). In the current legislation, labelling of vitamins and minerals for food products is not mandatory, and the passing of such information is voluntary for manufacturers. In Norway, the NFSA has recommended the manufacturers of the macroalgae industry to label the macroalgae products with details regarding iodine content (50). Of the macroalgae products with the declared iodine content, the declared values were deviant far above the measurement uncertainty, and for 18 out of the 24 declared products, the iodine content was deviating by more than 50%. With such variable labelling, often lacking either iodine content, species or both, it is impossible for the consumer to evaluate the iodine content and food safety of the products.

Processing of the macroalgae products, such as blanching and boiling, has reduced the iodine content in seaweeds and kelp (51, 52). During processing, macroalgae absorb water, which is species dependant (53); however, a higher water content in the product will result in a dilution effect of the iodine concentration when given on a wet weight basis. Much of the apparent reduction in iodine content (on a wet weight basis) that we found is thus a result of a higher water content after processing, and hence, calculating iodine loss or retention should be performed on a dry weight basis (54). Of the 43 wholefood macroalgae products included in this study, 30 had preparation instructions, presented either as serving suggestions or as methods proposed in order to reduce the iodine content. Processing the macroalgae products according to the suggested preparation methods resulted in an iodine loss ranging from 11 to 89% on a dry weight basis for most of the products, where variations may be due to different water solubility of different iodine species (55). For eight of the products (of which two were fresh algae), the calculated dry weight iodine content was higher after processing, a somewhat unexpected finding. We cannot exclude the possibility of measurement uncertainties for post-processed samples due to a high-water content (56). Furthermore, it is possible that water-soluble compounds associated with the algae (such as mucus) were washed out during processing (51), resulting in a more concentrated post-processed sample. Even though the iodine content was mostly reduced after processing, the amount of iodine consumed may still be of concern. For sea spaghetti, which may be consumed in quite large amounts when prepared, the iodine content in the ready-to-eat, post-processed product varied from 2 to 15 μg/g, which would correspond to 100–750 μg in a portion of 50 g. A portion of 50 g of the prepared sugar kelp would give 2,900–8,000 μg iodine. The analysed broth was also very high in iodine for some products, and consuming a standard portion of soup (350 g) could result in a severely excessive iodine intake.

### Strengths and limitations

A strength of this study is the description of the iodine content in a relatively large and broad sample of commercial macroalgae products available for consumers. These data may be used for estimating the iodine intake from macroalgae products, as well as for risk–benefit assessments of inclusion of such products in the diet. Limitations include that this study provides results only for a limited time frame in a young and dynamic market. The identification of the macroalgae products was carried out during the autumn of 2019, and new products have probably entered the market since then. Furthermore, we were omitting restaurants and catering outlets selling macroalgae products. We selected macroalgae products based on availability of the products. However, we cannot verify that the samples are representative for the macroalgae products available on the market, as we have not been able to use retail data for selection.

In this study, samples were analysed as composite samples rather than individual samples. There are both strengths and limitations for analysing samples through pooling. Pooling reflects the general levels (similar to using the mean of individual samples); however, content in single samples will not be reflected (57). Another limitation of sampling is the number of subsamples included in each composite sample, which ideally should be calculated from the variability of the nutrient composition of the foodstuff to provide a mean with a reasonable level of confidence (57). However, such a calculation requires a mean and SD on the nutrient composition of the foodstuff, which were not available for macroalgae. Contaminants and heavy metals were not taken into consideration in this current article, and this will be an important topic for future research.

## Conclusion

We identified a wide variety of available products containing seaweed and kelp from different product categories, namely, wholefood macroalgae, different food products containing macroalgae and dietary supplements containing macroalgae. Many of the wholefood macroalgae products and the foods containing macroalgae would, with regular use, result in an iodine intake above the UL. The macroalgae containing supplements cannot be relied upon for providing the daily RI of iodine, as some had an iodine content far below the declared content. Furthermore, there were supplements that would result in an intake above the UL. Dietary macroalgae may be a source of iodine, especially for people excluding animal foods. However, as the content of iodine was highly variable, and the labelling of the macroalgae-containing products was inadequate or inaccurate for several foods and supplements, inclusion of such products may pose a risk of consuming excessive amounts of iodine.

## Appendix 1

**Table:** Preparation according to manufacturer’s instructions.

Common name	Number	Product description	Preparation method (according to the description on the package)	Water samples^a^	Iodine content pre-processing		Iodine content post-processing	
					μg/g ww	μg/g dw	μg/g ww	μg/g dw
Nori	1	Dried	Washed and rinsed under a running tap.		15	16	2	24
Sea spaghetti	2	Dried	Soaked in cold water for 15 min. Washed and rinsed several times. Drained.		28	31	6,3	49
Sea spaghetti	3	Dried	Soaked in water for 15 min. Drained.		50	53	15	44
Sea spaghetti	4	Dried	Washed and soaked in lukewarm water for 30 min. Drained and rinsed before being added to fresh boiling water and simmered for 10 min.		72	77	2	28
Sea spaghetti	5	Dried	Soaked for 30 min. Drained.		50	56	4	32
Sugar kelp	9	Dried	Soaked in water for 25 min. Brought to a full boil and simmered for 10 min. Water sample extracted using a pipette. Drained.	Yes	1,900	2,120	59	474
Dulse	14	Dried	Washed and soaked in cold water for 10 min. Drained and rinsed briefly.		140	152	23	136
Oarweed	15	Dried	Soaked in cold water for 22.5 min. Simmered for 35 min. Water sample extracted using a pipette. Drained.	Yes	8,100	8,697	160	1,444
Oarweed	16	Dried	Soaked in water for 30 min. Drained.		7,500	8,545	140	901
Winged kelp	18	Dried	Soaked in cold water for 20 min. Simmered for 15 min in fresh boiling water. Drained.		190	216	4	54
Winged kelp	19	Dried	Rinsed under a running tap. Soaked in cold water for 5 min. Drained and brought to a full boil in freshwater. Simmered for 5 min. Drained and rinsed under a running tap.		190	215	9,7	159
Wakame	28	Dried	Soaked in water for 3.5 min. Drained. Added freshwater and brought to a full boil. Water sample extracted using a pipette. Drained.	Yes	140	152	2,5	70
Wakame	30	Dried	Water containing the macroalgae was brought to a full boil, then simmered for 5 min. Using a pipette, water was extracted before draining the macroalgae using a sieve.	Yes	260	294	17	186
Arame or sea oak	31	Dried	Washed and soaked in cool water for 12.5 min. Drained and rinsed in water.		400	434	33	213
Bladder wrack	34	Dried	Soaked in water for 25 min. boiled for 10 min. Drained.		120	137	4	23
Toothed wrack	35	Dried	Soaked in water for 25 min before being brought to a boil/simmering for 10 min.		250	271	50	182
Toothed wrack	36	Dried	Soaked in lukewarm water for 5 min.		190	221	11	77
Kombu	38	Dried	Soaked in water for 25 min. Brought to a full boil and simmered for further 5 min. Water sample extracted using a pipette. Drained.	Yes	1,200	1,273	NA	NA
Irish moss or ceregeen moss	43	Dried	Soaked in water for 4 h. Drained and rinsed well under a running tap.		NA	0	16	139
Wakame	44	Dried	Brought to a full boil and simmered for 5 min.		200	216	19	244
Wakame	71	Dried rice noodles with wakame (1%)	Boiled in water until al dente. Drained.		0,93	1	0.2	0.7
Wakame	83	Dried	Washed and soaked in lukewarm water for 12.5 min. Drained. rinsed and cooked for 5 min in fresh water. Water sample extracted using a pipette. Drained.	Yes	170	183	11	217
Kombu	84	Dried^b^	Soaked in water for 3.5 min. Drained.		2,600	2,704	NA	NA
Wakame	89	Dried	Soaked in water for 10 min. Drained.		92	98	9,3	120
Wakame	91	Soup sachet with wakame (5%)	Boiling water was added to sachets and stirred before extracting the water sample using a pipette.	Yes	5	5	NA	NA
Nori	92	Dried	Brought to a full boil and drained.	Yes	21	24	2	29
Kombu	93	Dried	Soaked in water for 25 min. Brought to a full boil and simmered for further 5 min. Water sample extracted using a pipette. Drained.	Yes	3,000	3,257	88	813
Sugar kelp	210	Fresh preserved with salt	To desalt. it was rinsed under a running tap for 2.5 min.		300	836	160	1,406
Sea spaghetti	211	Fresh preserved with salt	To desalt. it was rinsed under a running tap for 2.5 min.		5	12	7	52
Mix	212	Dried	Soaked in water for 4 min. Drained and rinsed.		220	245	19	197

Ww = wet weight. Dw = dry weight.

a Using a pipette, samples were extracted from water in which the macroalgae was prepared (i.e. the stock or broth) following preparation.

b The species had not yet been identified during preparation and was thus prepared in the same way as a wakame wholefood product.

## Appendix 2

**Table:** Portion sizes used to calculate the iodine content of one portion, and its contribution to recommended intake (RI) and upper tolerable intake level (UL).

Species	*n*	Mean	SD	Portion	μg/ per portion	% of RI (150 μg)	Times UL (600 μg)
Irish moss or carageen moss	1	16		8 g	128	85	0.2
Nori	2	18	4	8 g	144	96	0.2
Dulse	3	96	47	8 g	768	512	1.2
Wakame	5	172	63	8 g	1,376	917	2.3
Sea spaghetti	6	42	23	8 g	336	224	0.6
Bladder wrack	1	120		8 g	960	640	1.6
Arame or sea oak	1	400		8 g	3,200	2,133	5.3
Winged kelp	5	552	402	8 g	4,416	2,944	7.4
Toothed wrack	2	220	42	8 g	1,760	1,173	2.9
Mix	4	740	648	8 g	5,920	3,947	9.9
Truffle seaweed	1	1,400		8 g	11,200	7,467	18.7
Kombu	3	2,267	945	8 g	18,136	12,091	30.2
Sugar kelp	4	4,400	5,222	8 g	35,200	23,467	59
Oarweed	2	7,800	424	8 g	62,400	41,600	104.0

Foods with macroalgae	*n*	Mean	SD	Portion	μg/ per portion	% of RI (150 μg)	Times UL (600 μg)

Nuts	2	25	27	20^a^	500	333	0.8
Soup	1	5		350^b^	1,750	1,167	2.9
Breading	2	1,265	1,747	20^c^	25,300	16,867	42.2
Meat substitutes	2	1.2	1	150^d^	180	120	0.3
Chocolate	3	31	48	100^e^	3,100	2,067	5.2
Salt	4	588	594	6^f^	3,528	2,352	5.9
Seasoning blends	5	368	634	6^f^	2,208	1,472	3.7
Caviar	3	1	0	30^g^	30	20	0.1
Marinated or pickled seaweed	4	11	20	100^h^	1,100	733	1.8
Pesto	2	47	4	18^i^	846	564	1.4
Rice noodles with seaweed	1	1		85^j^	85	57	0.1
Crisps	2	11	13	24^k^	264	176	0.4
Nori-snack	4	25	7	8^l^	200	133	0.3
Blended snack	3	17	7	8^l^	136	91	0.2
mustard	1	6		16^m^	96	64	0.2

a One handful (1);

b One portion (1);

c Two measuring spoons of Semolina (1);

d One serving portion of meat (chicken/ beef) (1);

e One bar;

f Recommended daily amount not to be exceeded (2);

g Topping for two slices of bread (1);

h One dl of sauerkraut (1);

i One tablespoon (1);

j One portion noodles without eggs. dry (1);

k Two dl (1);

l One portion of dried seaweed (3);

m One table spoon (1).

1. Dalane JØ. Bergvatn TAM. Kielland E. Carlsen MH. Mål. vekt og porsjonsstørrelser for matvarer [Weights. measures. and portion sizes for foods] (in Norwegian). Oslo: Norwegian Food Safety Authority, University of Oslo, Norwegian Directorate of Health; 2015.
2. NNR. Nordic nutrition recommendations 2012: integrating nutrition and physical activity. Copenhagen: Nordisk Ministerråd; 2014. Report No.: 978-92-893-2670-4 (ISBN). 09037004 (ISSN) Contract No.: 2014:002.
3. MacArtain P, Gill CIR, Brooks M, Campbell R, Rowland IR. Nutritional value of edible seaweeds. Nutr Rev 2007; 65(12): 535–43. doi: 10.1301/nr.2007.dec.535-543

## Appendix 3

**Table:** Calculation of iodine content in one recommended dose of the different supplements, its contribution to recommended intake (RI) and upper tolerable intake level (UL).

Common name	Product number	Iodine (ug/g)	Recommended dose^a^	Dose in gram	μg/ per portion	% of RI (150 μg)	Exceeding UL (600 μg)
Rockweed	73	450	3 Capsules	1.8	810	540	1.4
Rockweed	79	290	1 Capsule	0.4	116	77	0.2
Rockweed	82	560	2 Tablespoons	10	5,600	3,733	9.3
Bladderwrack	77	360	1 Capsule	0.4	144	96	0.2
Bladderwrack	95	230	1 Capsule	0.34	78.2	52	0.1
Oarweed	78	8,3	2 Capsules	0.56	4.648	3	0.0
Giant kelp	74	160	4 Tablets	0.25	40	27	0.1
Sea lettuce	213	54	10 g	10	540	360	0.9
Sea lettuce	214	31	10 g	10	310	207	0.5
Rockweed, Bladderwrack	76	320	1 Tablet	0.28	89.6	60	0.1
Rockweed, Bladderwrack	96	150	2 Tablets	0.21	31.5	21	0.1
Rockweed, Wakame	75	27	3 Tablets	0.45	12.15	8	0.0
Rockweed, Bladderwrack, Toothed Wrack	81	320	250 mg/ tip of a knife	0.25	80	53	0.1
Not identified	205	460	1 Tablet	0.14	64.4	43	0.1

a Recommended by a manufacturer.
